# Bispecific antibodies—effects of point mutations on C_H_3-C_H_3 interface stability

**DOI:** 10.1093/protein/gzac012

**Published:** 2022-12-05

**Authors:** Nancy D Pomarici, Monica L Fernández-Quintero, Patrick K Quoika, Franz Waibl, Alexander Bujotzek, Guy Georges, Klaus R Liedl

**Affiliations:** Institute of General, Inorganic and Theoretical Chemistry, and Center for Molecular Biosciences Innsbruck (CMBI), University of Innsbruck, Innrain 80-82, A-6020 Innsbruck, Austria; Institute of General, Inorganic and Theoretical Chemistry, and Center for Molecular Biosciences Innsbruck (CMBI), University of Innsbruck, Innrain 80-82, A-6020 Innsbruck, Austria; Institute of General, Inorganic and Theoretical Chemistry, and Center for Molecular Biosciences Innsbruck (CMBI), University of Innsbruck, Innrain 80-82, A-6020 Innsbruck, Austria; Center for Protein Assemblies (CPA), Department of Physics, Chair of Theoretical Biophysics, Technical University of Munich, Ernst-Otto-Fischer-Str. 8, 85748, Garching, Germany; Institute of General, Inorganic and Theoretical Chemistry, and Center for Molecular Biosciences Innsbruck (CMBI), University of Innsbruck, Innrain 80-82, A-6020 Innsbruck, Austria; Roche Pharma Research and Early Development, Large Molecule Research, Roche Innovation Center Penzberg, Nonnenwald 2, Penzberg, 82377, Germany; Roche Pharma Research and Early Development, Large Molecule Research, Roche Innovation Center Penzberg, Nonnenwald 2, Penzberg, 82377, Germany; Institute of General, Inorganic and Theoretical Chemistry, and Center for Molecular Biosciences Innsbruck (CMBI), University of Innsbruck, Innrain 80-82, A-6020 Innsbruck, Austria

**Keywords:** bispecific antibodies, CH3-CH3 interface, dissociation mechanism, interface stability, molecular recognition

## Abstract

A new format of therapeutic proteins is bispecific antibodies, in which two different heavy chains heterodimerize to obtain two different binding sites. Therefore, it is crucial to understand and optimize the third constant domain (C_H_3-C_H_3) interface to favor heterodimerization over homodimerization, and to preserve the physicochemical properties, as thermal stability. Here, we use molecular dynamics simulations to investigate the dissociation process of 19 C_H_3-C_H_3 crystal structures that differ from each other in few point mutations. We describe the dissociation of the dimeric interface as a two-steps mechanism. As confirmed by a Markov state model, apart from the bound and the dissociated state, we observe an additional intermediate state, which corresponds to an encounter complex. The analysis of the interdomain contacts reveals key residues that stabilize the interface. We expect that our results will improve the understanding of the C_H_3-C_H_3 interface interactions and thus advance the developability and design of new antibodies formats.

## Graphical Abstract

**Figure:**
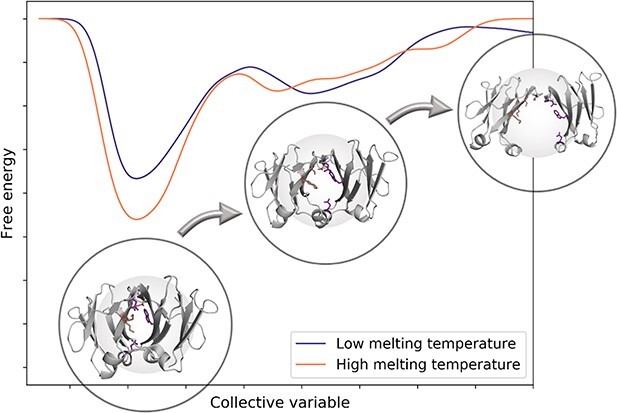

## Introduction

Monoclonal antibodies (mAbs) emerged in the pharmaceutical industry in the late 90s, and since then, more than a hundred antibodies have been approved (Kaplon *et al.*, 2020). In recent years, scientific advances led to substantial improvements in the development of mAbs (Carter, 2006). Current strategies focus on engineering antibodies by modifying their size or changing the number of antigen binding sites, leading to new formats as single-chain variable fragments, diabodies or nanobodies (Wang *et al.*, 2019). In particular, bispecific antibodies have been designed to bind two different antigens, representing a key component of the next generation of biotherapeutics (Glockshuber *et al.*, 1990; Holliger *et al.*, 1993; Spiess *et al.*, 2015; Yang and Shah, 2020). They offer several advantages, like blocking two different signaling pathways simultaneously and a potential to display activity that is not present in any combination of the parent antibodies (Shiraiwa *et al.*, 2019). As the half-life of antibodies relies on the FcRn, some bispecific formats aim to keep the Fc fragment combined with two Fabs or two other binding domains fused on Fc (Charles A Janeway *et al.*, 2001; Shiraiwa *et al.*, 2019).

The classical IgG structure is composed of 12 domains, sharing a β-immunoglobulin fold. There are two antigen-binding fragments (Fabs) containing four domains each that are bound to the crystallizable fragment (Fc), which defines the isotype and subclass of the immunoglobulin (Charles A Janeway *et al.*, 2001). The role of the Fc fragment, apart from the FcRn recycling, is to mediate the immune response, binding to the receptor on effector cells and to activate other immune mediators (Schroeder and Cavacini, 2010; Torres and Casadevall, 2008). Most IgGs assemble starting with a dimerization of identical heavy chains, which is primarily mediated by the C_H_3 domains, followed by the hinge formation via two disulfide bridges between the two heavy chains and by the covalent addition of light chains via disulfide bond formations (Feige *et al.*, 2010). The manufacturing of bispecific antibodies is quite challenging because they require heterodimerization of the two heavy chains, which have to bind to the correct light chains, to obtain two different binding sites. Several solutions have been implemented to favor the pairing of the desired light chain with the corresponding heavy chain (Brinkmann and Kontermann, 2017; Krah *et al.*, 2018). In a very first instance, it is fundamental to characterize and understand the dimerization process and the respective interactions that are responsible for stabilizing the C_H_3-C_H_3 interface.

The C_H_3 domains, similar to other IgG domains, consist of ≈100 amino acids arranged into two antiparallel β-sheets. The intrachain disulfide bridge is not required for the right folding and association to a dimer, but the oxidized state has a higher thermodynamic stability than the reduced one (Thies *et al.*, 2002). The formation of non-covalent interactions between the two C_H_3 domains is the first step of the assembly (Feige *et al.*, 2010). In contrast, the relatively slow dissociation of the C_H_3 domains is a kinetic barrier in the IgG4-arm exchange process, which leads to the formation of asymmetric, bispecific antibodies (Rispens *et al.*, 2011). In fact, previous studies showed that the C_H_3-C_H_3 is a highly stable homodimer with a dissociation force much higher than other studied protein–protein complexes (Bertz *et al.*, 2013). Nevertheless, the mechanism of dissociation has not been completely characterized yet and a structural and biophysical understanding of the process is crucial to enhance the developability of antibodies.

The development of bispecific antibodies improved dramatically with a specific design of the heavy chain interface to obtain heterodimers. Nowadays, the most widely used strategies for a successful heterodimerization are the knobs-into-holes (KiH) technology or charged modifications (Gunasekaran *et al.*, 2010; Ridgway *et al.*, 1996). The KiH approach is based on replacing a small amino acid with a bulky residue on one chain to create a ‘knob’, and introducing small residues on the other chain to obtain a ‘hole’ (Atwell *et al.*, 1997; Merchant *et al.*, 1998). Instead, the charge inversion approach modifies the electrostatic interface profile (De Nardis *et al.*, 2017). These techniques lead to a good yield and a successful heterodimerization of the domains, but sometimes physicochemical properties, such as stability, can be affected (Ma *et al.*, 2021; van Gils and Sanders, 2017). Therefore, it is necessary to evaluate the impact of the mutations on the overall interface stability and on the proper assembly of the two domains.

To resolve these open questions, we performed molecular dynamics (MD) simulations of 19 C_H_3-C_H_3 structures and compared their dynamics during the dissociation process. We chose this set of antibodies because of the availability of crystal structures and experimentally determined melting temperatures. The sequences in the dataset have been previously engineered by introducing point mutations in a homodimeric interface (Matsumiya *et al.*, 2011), following different approaches, i.e., charged inversion mutations (Choi *et al.*, 2015; De Nardis *et al.*, 2017; Gunasekaran *et al.*, 2010; Leaver-Fay *et al.*, 2016), KiH technology (Elliott *et al.*, 2014; Von Kreudenstein *et al.*, 2013) and computationally or rationally designed mutants (Leaver-Fay *et al.*, 2016). Moreover, we modelled a C_H_3-C_H_3 dimer inserting point mutations that are present in a monomeric Fc molecule, as example of a system that exists in the unbound state (Ying *et al.*, 2014). We enforced the domain dissociation using metadynamics (Barducci *et al.*, 2011). This allowed us to capture the dissociation process of the respective interfaces and to identify the key structural elements that mainly contribute to the interface stability.

## Methods

### Dataset

**Fig. 1:**
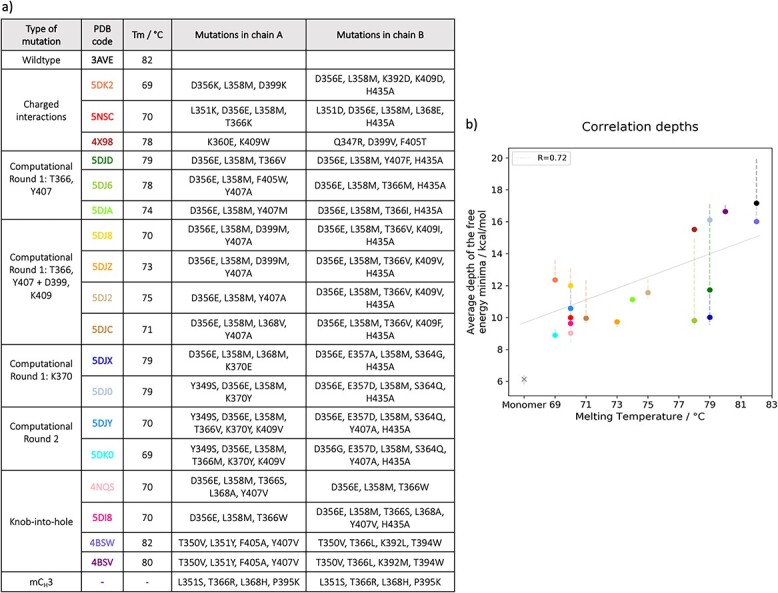
Correlation between depths of the free energy minima and melting temperatures. **a)** Table showing the PDB codes of the 20 systems with respective melting temperatures. Additionally, the point mutations that are present in each system are listed. **b)** The points, color coded according to the colors of the PDB colors in the table, represent the average depth of the free energy minima for each system. The error bars show the minimum and maximum of the average from the Leave-One-Out cross-validation. mC_H_3 is indicated by an ‘x’. No experimentally measured Tm is available for this variant; therefore, it is excluded from the correlation.

The dataset contains 19 crystal structures from the PDB database (Berman *et al.*, 2000). One of them is the wildtype, (PDB code: 3AVE (Matsumiya *et al.*, 2011)) and 18 are heterodimeric variants of the IgG1. A detailed list of all systems is present in Fig. 1a, with information about their experimentally measured melting temperatures and the respective point mutations present in chain a and chain b. The dataset is divided in subgroups depending on the type of point mutations that have been applied to reach heterodimerization: three of them are obtained with the mutation of charged residues (PDB codes: 5DK2 (Gunasekaran *et al.*, 2010; Leaver-Fay *et al.*, 2016), 5NSC (De Nardis *et al.*, 2017), 4X98 (Choi *et al.*, 2015)), 11 derive from computationally and rationally designed techniques (PDB codes: 5DJD, 5DJ6, 5DJA, 5DJ8, 5DJZ, 5DJ2, 5DJC, 5DJX, 5DJ0, 5DJY, 5DK0) (Leaver-Fay *et al.*, 2016), and other four are following the KiH technology (PDB codes: 4NQS (Elliott *et al.*, 2014), 5DI8 (Leaver-Fay *et al.*, 2016), 4BSW, 4BSV (Von Kreudenstein *et al.*, 2013)). To annotate the different homo-and-heterodimers, we used the EU numbering scheme (Edelman *et al.*, 1969). As an example of system that exists in solution in the unbound state, we introduced point mutations in the wildtype (PDB code: 3AVE (Matsumiya *et al.*, 2011)) interface that are present in a monomeric Fc (mFc) (Ying *et al.*, 2014) using MOE (Molecular Operating Environment, Chemical Computing Group, version 2018.01). In this paper, we will refer to this dimer naming it ‘mC_H_3’.

### Structure preparation

The Fc crystal structures were cut to obtain only the C_H_3-C_H_3 domains with 100 residues in chain a and 100 residues in chain b. The starting structures for simulations were prepared in MOE (Molecular Operating Environment, Chemical Computing Group, version 2018.01) using Protonate3D (Labute, 2009). To test the role of the C_H_2 domains in stabilizing the C_H_3-C_H_3 dimer, we ran the protocol also for the complete Fc of one KiH system (PDB code: 4NQS) (Elliott *et al.*, 2014).

### Molecular dynamics simulations of bound state

Using tleap of the AmberTools19 package (Case *et al.*, 2005; Salomon-Ferrer *et al.*, 2013a), the crystal structures were solvated in cubic water boxes of TIP3P water molecules with a minimum wall distance of 10 Å to the protein (Jorgensen *et al.*, 1983). The AMBER force field ff14SB was used (Maier *et al.*, 2015). Each system was equilibrated using a multistep equilibration protocol. First hydrogen atoms and water molecules are minimized using 500 steps of steepest descent and 500 steps of conjugate gradient. Then the system was heated from 100 to 300 K with a 200 ps NVT simulation, using a time step of 1 fs and a Langevin thermostat. Finally, 200 ps of NPT simulation was performed using a Berendsen barostat at 300 K to equilibrate the pressure (Wallnoefer *et al.*, 2011).

We performed 1 μs of conventional Molecular Dynamics (cMD) simulations on the equilibrated structures, to characterize the bound state of the C_H_3-C_H_3 domains. The simulations were performed in an NpT ensemble using the CUDA implementation of the pmemd program (Salomon-Ferrer *et al.*, 2013b). Bonds involving hydrogen atoms were restrained using the SHAKE algorithm, with a time step of 2.0 fs (Miyamoto and Kollman, 1992). We used the Berendsen algorithm to keep the system at atmospheric pressure (1 bar) (Berendsen *et al.*, 1984). The temperature during the simulations was maintained at 300 K using the Langevin thermostat (Adelman and Doll, 1976). The cutoff for non-bonded interactions was set to 8 Å.

We calculated the fraction of native contacts in relation to the native structure and the distance between the centers of mass (COM) of the two C_H_3 domains for the resulting trajectories of each system using cpptraj (Roe and Cheatham, 2013).

### Metadynamics simulations

Since a dissociation of the two domains is expected during the metadynamics simulation, we solvated the crystal structures in larger cubic water boxes (25 Å of minimum wall distance to the protein). In this way, we ensure that no interactions of the protein with the periodic images take place during dissociation. Again, the systems were equilibrated following a multistep equilibration protocol, as described above (Wallnoefer *et al.*, 2011). We used enhanced sampling to enforce the dissociation process (Bussi and Laio, 2020). Metadynamics (Barducci *et al.*, 2011) simulations were performed in GROMACS (Abraham *et al.*, 2015; Pronk *et al.*, 2013) with the PLUMED 2 implementation (Tribello *et al.*, 2014). Like other enhanced sampling techniques, metadynamics requires the selection of suitable low dimensional collective variables (CVs), which can describe the process of interest (Barducci *et al.*, 2011; Bussi and Laio, 2020). As CV, we chose the distance between the centers of mass (COMs) of the domains. The simulations were performed in the NpT ensemble with a pressure control guaranteed by the Parrinello-Rahman barostat. The Gaussian deposition occurred every 5000 steps and had a height of 0.1 kJ/mol and a width of 0.05 nm. About, 10 runs of 80 ns of metadynamics simulations were performed for every system. Metadynamics allows us to induce transitions to metastable states, but also to derive the free energy change of complex molecular processes (Bussi and Laio, 2020). For each simulation, we computed the free energy surface along the collective variable (Laio and Parrinello, 2002).

### Description of the dissociation

We were interested in a description of the dissociation process, i.e., the transition of the system from the bound to the dissociated state. Thus, we used the descriptors explained below to define bound and dissociated states and considered everything else to be in the process of dissociation. We call these states encounter complexes.

### Definition of the dissociation states

The free energy can be estimated as a function of the metadynamics CV, with the PLUMED 2 implementation (Tribello *et al.*, 2014). The difference in free energy between the minimum corresponding to the bound state and the transition region towards the unbound state provides an estimate of the relative stability of the C_H_3-C_H_3 structures. The time in which the dissociation starts can be defined by considering the bound state obtained from the cMD trajectories as a reference point. The mC_H_3 is the only system that dissociates already within the 1 μs cMD simulation. Therefore, we only considered the first 600 ns of the cMD trajectory, because until this point, we observe no big shifts in the descriptors’ timeseries. The values of the fraction of native contacts and the distance of centers of mass during the cMDs exhibit a bell-shaped distribution. A decreasing sigmoidal function can be fitted on the right side of the bell curve of the distance, since the distance increases during the dissociation process. In the same way, a sigmoidal function can be fitted on the left side of the bell curve of the fraction of native contacts, because the dissociation causes a loss of native contacts (Supplementary Fig. 1a). The parameters (slope and turning point) obtained by the fitting procedure are then used to project a sigmoidal function on the timeseries of distance and fraction of native contacts during the metadynamics simulations (Supplementary Fig. 1b). The equation of the sigmoidal function is

$$ \frac{1}{1+{e}^{-s\ast \left(x-x0\right)}} $$

where *s* represents the slope of the curve and *x0* the turning point.

The multiplication of the two sigmoids results in the state definition. State 1 corresponds to the bound state, and it smoothly approaches 0, which represents instead the dissociated state. The state definition can be applied to identify in each metadynamics simulation the point in time in which the structure is dissociating (Supplementary Fig. 1c).

The free energy curves are estimated until the starting point of dissociation of each simulation and, therefore, only show the minimum corresponding to the bound state. Afterwards, we computed the 10 free energy curves, with respect to the underlying probability distributions, separately. According to the Boltzmann distribution, we obtained probability density functions from the resulting free energies. Furthermore, the weight of each trajectory is calculated from the time dependent bias c(t) (Tiwary and Parrinello, 2015). We combined the probability distributions of the individual simulations, multiplying them with the respective weights, and renormalized the obtained probability distribution. Then, we performed a Boltzmann inversion of the resulting probability distribution to obtain the corresponding free energy space. The depth of the minimum was calculated, and the uncertainty was estimated with the leave one out cross validation method. The analysis of the metadynamics trajectories have been performed taking into account the so-calculated weights.

Following a similar procedure, the transition from the encounter complex to the unbound state is defined using as descriptors the fraction of native contacts and the solvent accessible surface area (SASA). Not only they can describe the process of interest, but also their timeseries plot during the metadynamics simulations reaches a plateau when the structure is completely dissociated. Also in this case, a sigmoidal function can be fitted on both timeseries (Supplementary Fig. 1d) and afterwards the two curves are normalized and multiplied together. In this new state definition curve, state 1 corresponds to the bound state and state 0 corresponds to the completely dissociated state (Supplementary Fig. 1e). The point at which the state definition curve approaches zero is considered as the start of the unbound state.

### Molecular dynamics simulations of the cluster representatives

We chose the homodimer 3AVE (Tm = 81 °C) for further simulations to describe the dynamics and thermodynamics of dissociation. We combined the 10 resulting metadynamics trajectories, considering only the bound state and the encounter complex. Then, we performed an average linkage hierarchical clustering in cpptraj (Roe and Cheatham, 2013), using as distance metric the root mean-square deviations (RMSD) values of the C_H_3-C_H_3 atoms after alignment of the structure on chain a, with a distance cutoff criterion of 0.1 Å, resulting in 93 clusters. The cluster representatives were solvated in a cubic water box with a minimum wall distance to the protein of 20 Å and equilibrated using the same protocol as before. We ran 200 ns cMD simulations using the AMBER20 simulation package, following the same protocol that has been previously described (Case *et al.*, 2020).

We then performed a time-lagged-independent component analysis (tICA) on the obtained trajectories, using the Python library PyEMMA 2, employing a lag time of 10 ns (Scherer *et al.*, 2015). TICA identifies the slowest degrees of freedom of the system. As features, we used the inverse distances between the CA atoms of the residues that make contact in the interface of the first frame of the metadynamics simulations. The types of contacts that are considered are: hydrogen bonds, salt bridges, pi-cation, pi-stacking, aromatic T-stacking and hydrophobic contacts. The inverse distances emphasize changes at small distances and filter out changes that take place at large distances. The features applied to build each tICA are listed in the Supplementary Material (Supplementary Table 1).

We obtained the thermodynamics and kinetics from a Markov-state model (MSM) (Chodera and Noé, 2014), which is based on a k-means clustering algorithm to define microstates (in our case 90) and the PCCA+ clustering algorithm to coarse grain them into macrostates (Likas *et al.*, 2003). MSM is a powerful tool to predict the long time-scale dynamics of molecules, starting from a pool of short MD simulations (Da *et al.*, 2014). The transitions reflect the free energy surface and the dynamics of the system. The sampling efficiency and the reliability of the MSM is evaluated by the Chapman–Kolmogorov test (Supplementary Fig. 2b), assuring that the network states are fully connected, which is a necessary requirement to calculate probabilities of transitions (Miroshin, 2016). We chose a lag of 9 ns since the computed relaxation timescales at this lag time are approximately constant (Supplementary Fig. 2a). We then used the respective macrostate ensembles to investigate the relative orientation of the domains during the dissociation process.

### Interface contact analysis

The GetContacts program is a useful tool to compute atomic interactions in protein structures (https://getcontacts.github.io/). It shows how the contacts between the residues evolve during the simulation, and which type of contacts occur. We calculated the overall number of contacts, which include hydrogen bonds (backbone/backbone, side chain/backbone and side chain/side chain), salt bridges, pi-cation, pi-stacking, T-stacking and hydrophobic contacts. Van der Waals interactions, which are only defined based on the distance between the residues, have been excluded from the analysis. The contacts are defined using the default geometrical criteria recognized by GetContacts. The contacts in the bound and dissociated structure are calculated concatenating the respective parts of the 10 repetitions for one system together. We described the mechanism of dissociation using the so-called flareplots, in which we coarse-grained the residues that belong to the same loops or β-strands. The secondary structure has been assigned using STRIDE (Supplementary Fig. 3) (Frishman and Argos, 1995).

### Interdomain orientation

The mutual orientation between the domains is calculated using the OCD tool (Hoerschinger *et al.*, 2021). This tool extends the previous ABangle approach (Dunbar *et al.*, 2013) to characterize the interdomain orientation not only in the Fv region and TCRs, but also in the constant domains. It creates a suitable coordinate system based on a user-defined reference structure. The orientation relative to this reference is described by two tilt angles for each vector towards the center axis (AC1, AC2, BC1, BC2), the length of the center axis (dC) and a torsion angle (AB) between the two intersecting planes composed of A1, the center axis and B1.

## Results

We performed metadynamics simulations on the dataset presented in Fig. 1a to obtain information about the dynamics of the systems during the dissociation process and to explore the role of point mutations on interface stability.

### Correlation between depths of the free energy minima and melting temperatures

As described in the Methods section, ten runs of metadynamics simulations were performed for each system. We estimated the free energy curves from the metadynamics simulations until the starting point of dissociation for the 10 repetitions, then we normalized and averaged them together. We find a good correlation between the depth of the free energy minima and the experimentally determined melting temperatures (Fig. 1b). We estimated the errors with the Leave-One-Out cross validation. mC_H_3 is represented in the plot with a different symbol (‘x’) because it is excluded from the correlation, since no experimental data are available. Its instability suggested by its existence as a monomer in solution is confirmed here by the lowest free energy associated to its bound state.

We performed the protocol described in the Methods section of the Fc of the KiH system with the PDB code 4NQS to show the effect of the elimination of the C_H_2 domains on the free energy of the structure (Supplementary Fig. 4). We then calculated the average depth of the free energy minima and compare it with the one derived from the simulations on the dimer only. The values of free energy are comparable; therefore, we can assume that the exclusion of the C_H_2 domains does not dramatically influence our free energy results.

### Characterization of the bound ensemble

**Fig. 2:**
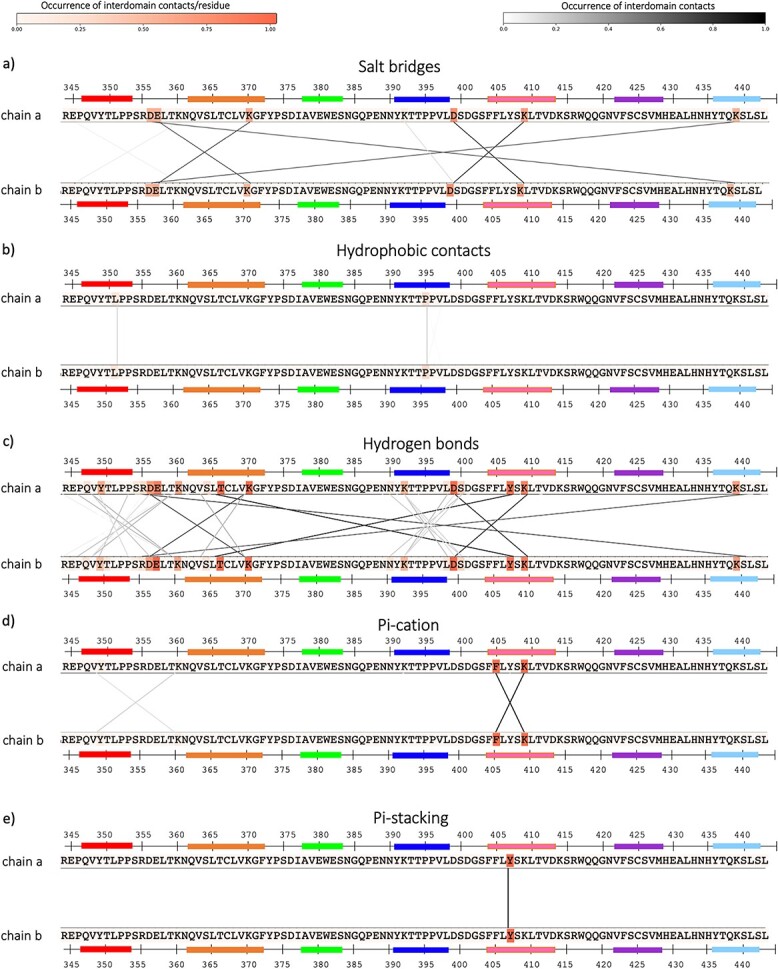
Contacts in the bound wildtype structure. The contacts between chain a and chain b in the metadynamics simulations are calculated until the dissociation point. The residues in chain a and b (in this case, the sequences are the same because it is a homodimer) are colored in different shades of orange according to the occurrence of contacts that they make. The residues that make contacts are connected by lines, colored in different shades of grey according to their occurrence.

We calculated the contacts that are present in the bound interface during the metadynamics simulations. We chose the homodimer wildtype (PDB code: 3AVE), to provide an exemplary representation of the interdomain contacts (Fig. 2). The other systems share similar patterns as they only differ in point mutations. The residues of the wildtype sequence are colored according to the occurrence of interdomain contacts that happen during the simulation (before dissociation). The residues that make contacts are connected by lines, which are also colored according to the occurrence of the contact. As expected, most of the contacts are hydrogen bonds (Fig. 2c). A relevant hydrogen bond is the one between T366 and Y407 (EU numbering scheme (Edelman *et al.*, 1969)) in both chains. The computationally and rationally designed heterodimers often include the mutation T366V (PDB codes: 5DJD, 5DJ8, 5DJZ, 5DJ2, 5DJC, 5DJY), T366M (PDB codes: 5DJ6) or T366I (PDB codes: 5DJA), and in these cases, the critical hydrogen bond T366-Y407 is missing. We find generally less hydrophobic contacts compared to the electrostatic interactions (Fig. 2a**and** b). Two residues, namely F405 and K409, are situated in the center of the interface in both chain a and chain b and form pi-cation interactions (Fig. 2d). Additionally, we find a pi-stacking interaction between the residues Y407-Y407 in chain a and in chain b (Fig. 2e). These interactions are present in all the systems, if these residues stay unmutated (Dall’Acqua *et al.*, 1998).

**Fig. 3:**
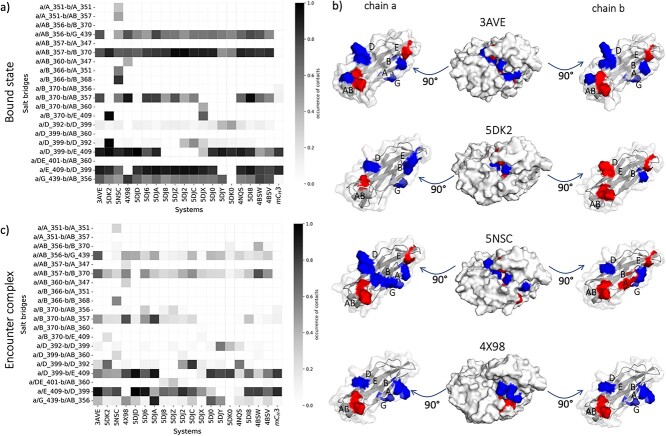
Comparison of salt bridges in the bound structures of the systems. **a)** Salt bridge occurrence between chain a and chain b of all the systems during the metadynamics simulations, until the start of the dissociation. The residues involved in a salt bridge are listed on the vertical axis of the matrix. The residue number is accompanied by a first letter (a or b), representing the chain, and a capital letter, representing the strand or loop in which the residue is located. **b)** The pictures show the open interface of the wildtype and of systems with charged mutations (PDB codes, respectively: 3AVE, 5DK2, 5NSC, 4X98). The residues in the interface that make salt bridges are colored: in blue the residues with a positive charge and in red the ones with a negative charge. **c)** Salt bridge occurrence between chain a and chain b when the systems are in the encounter complex states, colored according to their occurrence.

To monitor the changes in interactions within the dimers, we quantify the interdomain salt bridges and the hydrophobic contacts and compare them between the different bound structures. Figure 3a summarizes the differences in interdomain salt bridges between the different homo-and-heterodimers. Only the contacts with an occurrence higher than 20% are shown. Most of the systems show salt bridges at the same position as in the wildtype, e.g., between residues in position 409–399 (respectively in strand E and D), 370–357 (respectively in strand B and loop AB) and 356–439 (respectively in loop AB and strand G) in both chains. We find major differences in systems with charge mutations (PDB codes: 5DK2 and 5NSC). To visualize the position of these charged residues in the interface, both sides of the interface are displayed individually, and the surface of the charged residues is colored according to the occurrence of contacts that they make (Fig. 3b). The 4X98 contains two point mutations that introduce a new salt bridge (K360E in chain a and Q347R in chain b). However, the occurrence of this salt bridge is rather low (around 50%). The same comparison can be made for the encounter complexes (Fig. 3c), which correspond to the state in the metadynamics simulation between the bound and the unbound ones. In comparison, a regularly occurring salt bridge between residue K409 and D399 in both chains plays a central role also during the dissociation process. This salt bridge is also the only one that occurs with high occurrence in mC_H_3. In fact, this system has only few contacts between the A and B strands, which is already a sign of its high instability as dimer in solution.

By comparing the interdomain hydrophobic interactions between the systems of the dataset (Supplementary Fig. 5), we find less hydrophobic interactions compared to the salt bridge interactions: on average, the salt bridges are ≈29% of all the contacts that are present until dissociation, instead the hydrophobic interactions are only ≈10%. Some hydrophobic interactions can occur between F405 in chain a, when the residues 409 in the chain b is mutated to a hydrophobic one (e.g. K409I in 5DJ8 and K409V in 5DJZ and 5DJ2, K409F in 5DJC). Highly occurring hydrophobic contacts are instead present in the KiH variants (4NQS, 5DI8, 4BSW, 4BSV). In these four systems, a W is introduced in chain a or in chain b (at position 366 in the 4NQS and 5DI8 and at position 394 in the 4BSW and 4BSV, as shown in Supplementary Fig. 4a), to create a knob, which makes hydrophobic contacts with the non-bulky residues in the facing chain. Supplementary Fig. 5c shows the hydrophobic contacts in the encounter complexes. As already seen for the salt bridges, the occurrence of interactions decreases. Interestingly, the highest number of hydrophobic contacts during dissociation is present between the F405 in chain a and the residue in position 409 in chain b, in the systems in which this is mutated to a hydrophobic one (5DJ8, 5DJZ, 5DJ2 and 5DJC). Interestingly, mC_H_3 shows almost no hydrophobic interactions.

### Mechanism of dissociation

#### Interface contacts

**Fig. 4:**
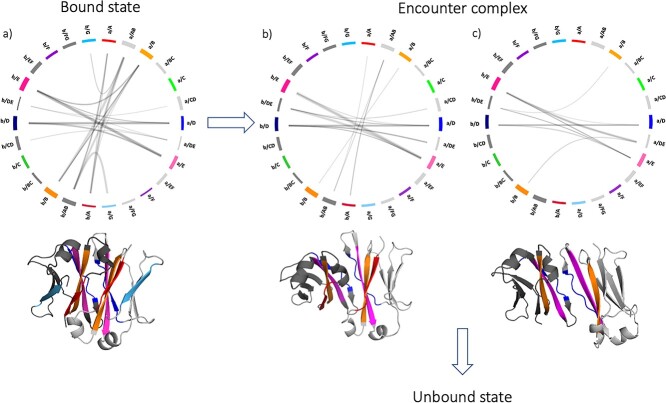
Mechanism of dissociation. The figure shows the change of the interface contacts during the dissociation process. The contacts between chain a and chain b are represented by flareplots in which the residues of the same secondary structure elements have been coarse grained. On the right side there are the elements of chain a, and on the left those of chain b. Next to each flareplot, a schematic representation of the structure in that point of dissociation is represented, in which the strands that make contacts are colored in accordance with the flareplot. The arrows indicate the different pathways in which the dissociation can progress.

We calculated the contacts during the metadynamics simulations to analyze their evolution over the dissociation process. We represent the contacts at the interface between chain a and chain b by flareplots in which the residues of the same secondary structure element have been coarse grained together, following the STRIDE annotation (Frishman and Argos, 1995) and colored according to Supplementary Fig. 3. Figure 4 shows the evolution of the interdomain contacts during the dissociation process. The wildtype has been chosen as an example to show the possible mechanisms of dissociation. Each flareplot shows the contacts that take place in a timeframe of 10 ns. The bound structures have contacts between all the β-strands and loops that shape the interface (A, AB, B, D, DE, E, G in both chains) (Fig. 4a). When the two domains dissociate, the first contacts that are lost are the ones between the loops AB and the facing β-strand G (Fig. 4b). From this point, the structure can directly evolve into an unbound state, or a further reorientation of the domains can take place, which results in a loss of the A strand contacts (Fig. 4c). The structure then dissociates completely.

#### Interdomain orientation

Using the OCD tool (Hoerschinger *et al.*, 2021), we describe the interdomain orientation with one distance (dC), one torsion angle (AB) and four bend angles (AC1, BC1, AC2, BC2). We obtained the standard deviation of each angle for each of the 10 repetitions of each system during the bound and encounter state, as a measure of variability. We averaged the 10 values of the standard deviation of the repetitions. The results are shown in Supplementary Table 2. In 17 out of 19 systems, the major variance is present in the AB angle, meaning that this angle dominates the dissociation process of C_H_3-C_H_3 dimers. This is also visible in Supplementary Fig. 6, which shows each angle plotted against the dC distance, from the merged trajectories for each system in the bound state and encounter complex. As expected, all the angles vary in the dissociation process, but the biggest variance can be seen in the AB angles. This indicates that torsions, i.e., rotation of the domains around the dC axis, are more pronounced than tilt movements.

### Kinetic and thermodynamic characterization of the dissociation process

**Fig. 5:**
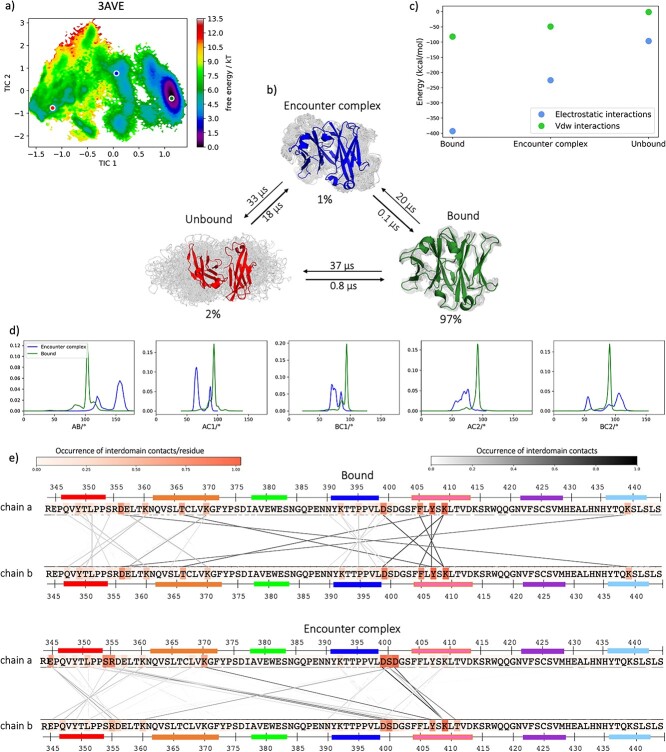
Kinetic and thermodynamics characterization of the dissociation process. The Markov state model allows to identify three states in the dissociation process: a bound state (green), an encounter complex (blue) and a dissociated state (red). **a)** TICA plot of the wildtype (PDB code: 3AVE). Three representatives are indicated with a dot, color-coded according to the dissociation state that it represents. **b)** Transition timescales between the macrostates. The structures represent the three different states of dissociation: the representative is highlighted in the front and the ensemble of possible structures is in the background. The probabilities of each state and the times for the transitions from one state to the other are reported. **c)** Comparison of the electrostatic and van der Waals energies between the three states. **d)** Distribution of OCD angles in the bound state and encounter complex. The biggest shift can be seen for the AB angle. **e)** Interdomain interactions in the bound state and in the encounter complex. The residues are colored according to the occurrence of the contacts that they make with the facing chain. The lines between the sequences connect the residues that make contacts, and they are also color-coded according to their occurrence.

For each metadynamics trajectory, we determined the point of the transition from the bound state to an encounter complex and from the encounter complex to the unbound state, based on three relevant descriptors: distance between the centers of mass, fraction of native contacts and SASA. The cMD simulations of each structure help to clearly define the bound state. Instead, when the fraction of native contacts between the domains is zero and the SASA reaches its maximum value, the structure can be considered completely dissociated. The state in between is the encounter complex. To better define the kinetics and the thermodynamics of this process, we ran unbiased simulations on representative structures of the three states of the wildtype (PDB code: 3AVE). To do this, we cut the 10 metadynamics trajectories considering only the bound state and the encounter complex and concatenated them. We clustered the resulting trajectories as described in the Methods section and used the cluster representatives as starting structures for each 200 ns cMD simulations. In this way, we can confirm that our findings are observable also in the resulting unbiased simulations and we can gain information regarding the kinetics and the thermodynamics of the dissociation process. Figure 5a shows the resulting tICA plot based on the inverse distances between the residues that make contacts in the first frame of the metadynamics simulations. Consistent with previous results, three macrostates can be identified, which correspond to the bound structure (represented in green), the encounter complex (blue) and the dissociated structure (red). Figure 5b illustrates the structural representation of the three states, showing a highlighted representative structure in the front and the ensemble of the possible structures of the respective state in the background. The bound state, which is also the most probable state, can transit to the other two states in the microsecond timescale. Moreover, we calculated the interaction energies at the interface for all obtained macrostates, with the LIE tool implemented in cpptraj (Roe and Cheatham, 2013), separating the electrostatic and the Van der Waals (VdW) contributions (Fig. 5c). The high stabilization of the interface in the bound state, especially by an electrostatic contribution, decreases during dissociation. Additionally, we calculated the angles of the respective macrostate ensembles of the bound state and the encounter complex with the OCD tool (Hoerschinger *et al.*, 2021), and their distribution is represented in Fig. 5d. The dissociated state is excluded in this analysis. Also in this case, we observe that the biggest shift between the bound state and the encounter complex can be seen for the AB angle. We also characterize the interdomain interactions in the two states before dissociation and we represent them in Fig. 5e. As previously shown with the flareplots (Fig. 4), most of the contacts appear between the β-strands A (red), B (orange), D (blue) and E (pink), while the β-strand G (light blue) is not involved in interdomain interactions anymore.

## Discussion

In this work, we compared the dissociation process of 19 C_H_3-C_H_3 crystal structures. In addition, we modelled a dimer, here named mC_H_3, in which we inserted the point mutations that characterize an Fc that exists only as monomer in solution (Ying *et al.*, 2014). C_H_3-C_H_3 domains have received strong interest in immunology and drug design because they are key to reach heterodimerization of the two heavy chains, which is fundamental for the production of bispecific antibodies. Several techniques have been developed to create heterodimers, which can then include two different binding sites (Choi *et al.*, 2013; Merchant *et al.*, 1998). The antibodies in our dataset were developed using a broad variety of techniques for heterodimerization. Point mutations have been inserted in a homodimer (3AVE) (Matsumiya *et al.*, 2011), following different strategies, such as modifications of charged interactions, rationally design or KiH mutations (Choi *et al.*, 2015; De Nardis *et al.*, 2017; Elliott *et al.*, 2014; Gunasekaran *et al.*, 2010; Leaver-Fay *et al.*, 2016; Von Kreudenstein *et al.*, 2013). Not only do these systems show high yields, but also experimentally measured melting temperatures are available. Figure 1a shows the melting temperatures and the point mutations that characterize each system in the dataset. In this work, we focus on the C_H_3-C_H_3 interface, as it is crucial for guiding new engineering efforts to develop novel antibodies format. Therefore, the C_H_2 domains present in the crystal structures of the systems in our dataset were removed and the whole protocol was performed on the C_H_3-C_H_3 dimers only. To verify the impact that this modification can cause on the free energy corresponding to the unbound state, the complete protocol has been applied also to the Fc of a KiH system (PDB code: 4NQS) and we compared the result with the corresponding C_H_3-C_H_3 dimer. This comparison reveals that the removal of the C_H_2 domains does not affect the average depth of the free energy minima (Supplementary Fig. 4).

The melting temperature is commonly used as a descriptor to quantify protein thermal stability. The complex network of protein interactions plays an important role in thermostability, and both attractive and repulsive interactions have to be considered to characterize this property (Codina *et al.*, 2019; Folch *et al.*, 2010). The unfolding of a mAb is a complex reaction with many intermediates (Garidel *et al.*, 2020). In this work, we consider the melting temperature as a descriptor of the domain dissociation process. In general, our computational result allows to distinguish between low and high thermal stable systems (Fig. 1b). mC_H_3 represents the lowest stable dimer in our dataset since it exists only as monomer in solution. This is confirmed by our computational results: on one hand, it is the only dimer that already dissociates in the unbiased cMD simulation, on the other, the average depth of the free energy minima is the lowest compared to the other systems (Fig. 1b). However, the free energy values corresponding to the depth of the bound state can differ between systems that have the same melting temperature. This is due to different reasons: first, all models are limited by the experimental errors present in the dataset and also the dissociation is not a complete description of the melting, which is instead a highly complex process. Moreover, the various interaction types that are present in the interface co-determine thermal stabilization. For example, distinct salt bridges may be differently affected by temperature changes and this can be reflected in the geometry of the interaction and the compactness of the structure (Folch *et al.*, 2008). On the other hand, hydrophobic effects seem to play a marginal role (Priyakumar, 2012).

We then characterized the conformational changes during the dissociation process and show how the point mutations can affect the interface stability. We observe three states during the process, a bound state, an encounter complex and a dissociated state, which are defined by comprehensive descriptors, such as distance between the centers of mass, fraction of native contacts and SASA.

Focusing on the interdomain contacts, the number and the occurrence of salt bridges at the interface between the two domains are much higher compared to the hydrophobic contacts (Fig. 2). This is in line with previous studies, which show that the C_H_3-C_H_3 interface is mainly characterized by salt bridge interactions, while the hydrophobic contacts are dominantly present in the structurally similar C_H_1-C_L_ domains (Fernández-Quintero *et al.*, 2022). The most occurrent salt bridges are present between the residues K409-D399, D356-K439 and E357-K370 in both chains, which surround the hydrophobic core. As expected, some differences are present in the systems with charge inversions, (PDB codes: 5DK2 and 5NSC) (Fig. 3). The mutations in position 399 in chain a and 392 in chain b (a_D399K, b_K392D, coexisting in the 5DK2 (Leaver-Fay *et al.*, 2016)) allow a salt bridge that occurs much more frequently than in the other systems. If the residue in position 409 in chain b is mutated to a D (b_K409D), it makes an additional salt bridge with K370. On the other side, the point mutation D356K in chain a in the 5DK2 introduces a repulsive interaction with the facing b_K439. The cMD simulation of this system shows a shift in the AB and AC1 angles, indicating a reorientation of the two domains. Consequently, the two positively charged residues are further apart, allowing water in between.

The 5NSC (De Nardis *et al.*, 2017), instead, shows not only charge inversions, but also additional mutations in which neutral residues are replaced by charged ones (i.e. a_L351K, a_T366K, b_L351D, b_I368E). This results in an increased number of charged contacts: in addition to the contacts that are also present in the wildtype, the K366 in chain a makes a salt bridge with the D351 and the E368 in the opposite chain, and the K351 in chain a has contacts with E357 and D351 in chain b. Nonetheless, the change of interdomain orientation during the dissociation process provokes a loss of some contacts, and in fact the salt bridges a_K351-b_E357 and a_K366-b_D351 are not present anymore in the encounter complex.

Further engineering of the C_H_3-C_H_3 interface resulted in a system in which charged residues mutations and KiH are combined (PDB code: 4X98 (Choi *et al.*, 2015)). In fact, in addition to charge inversions, a W has been introduced in chain a (a_K409W) and bulky residues in the opposite chain have been substituted with smaller ones to create a hole (b_D399V, b_F405T). Therefore, the system is stabilized not only by salt bridges, but also by hydrophobic interactions, which are instead missing in the 5DK2 and 5NSC (Supplementary Fig. 5). Indeed, this system has the highest melting temperature between the systems with charge mutations.

In the hydrophobic core of the structure, there are several other residues that interact in every frame of the simulations: F405 makes a pi-cation contact with K409, and the two tyrosines at position 407 are involved in a pi-stacking interaction. It has been shown that the residue in position 409 is relevant for the Fab-arm exchange process, and when it is a lysine, the resulting IgG is particularly stable (Labrijn *et al.*, 2011). Moreover, Y407 interacts with T366 by a hydrogen bond, which is highly occurrent during the simulations (Fig. 2). Our results agree with previous findings, which state the relevance of six residues for the stabilization of the C_H_3-C_H_3 interface (T366, L368, P395, F405, Y407 and K409) (Dall’Acqua *et al.*, 1998; Miller, 1990; Yang *et al.*, 2018).

As previously reported, the hydrophobic contacts in the C_H_3-C_H_3 interface are not numerous. By comparing how they differ between the systems of our dataset when the structures are still bound (Supplementary Fig. 5), we notice a higher number of hydrophobic contacts in the rationally designed systems, in which the K409 is mutated to a hydrophobic residue (respectively K409I in 5DJ8, K409V in 5DJZ and 5DJ2, and K409F in 5DJC). This allows the newly introduced residue to make a hydrophobic contact with F405. The biggest changes in hydrophobic contacts, though, are present for the systems that are engineered with the KiH technology (4NQS, 5DI8, 4BSW and 4BSV (Elliott *et al.*, 2014; Ridgway *et al.*, 1996)). In these cases, a W is inserted in chain b to interact with the facing hydrophobic cavity. The mutation happens in position 366 in the 4NQS system, and in position 394 in the 4BSW and 4BSV. The hydrophobic pocket is formed by the residues in position 368, 407 and 405 in chain a.

We also calculated the interdomain contacts in the encounter complexes. Both the salt bridges and the hydrophobic interactions occur less frequently. The most relevant salt bridge that keeps the interface together is the one between the residues in position 399 and 409 on both chains (Fig. 3c). Relevant point mutations for the dissociation process can be found in the systems 5DJ8, 5DJZ, 5DJ2 and 5DJC, in which the residue in position 409 in chain b is mutated to a hydrophobic one and it therefore makes quite frequent hydrophobic contacts with F405 also during dissociation (Supplementary Fig. 4c). On the other side, the loss of the positively charged K409 in chain b prevents the formation of the salt bridge with the residue in position 399 in the opposite chain. The substitution of the salt bridge with a hydrophobic contact has most likely a negative effect on the stability of the dimer, reflected in the lower melting temperatures that these systems have compared to the rest of the dataset. This is also confirmed by the low stable systems derived from the computational round 2 (PDB codes: 5DJY and 5DK0), in which the salt bridge a/E_409-b/D_399 is prevented by the mutation K409V.

In general, the contacts between the β-strands D and E play a central role to avoid a complete dissociation of the two domains. On the other hand, these contacts alone are not enough to keep the domains together. In fact, even if the salt bridge D_399/E_409 is the only occurrent interaction that takes place in mC_H_3, the structure is nevertheless completely dissociated in solution and highly unstable in our simulations. Contacts between the β-strands A and B and the loop AB are completely missing, as well as hydrophobic interactions. This causes an immediate dissociation of the structure and low stability of the dimer. Therefore, when engineering C_H_3-C_H_3 interfaces, the electrostatic interactions between the D and E strands have to be preserved to enhance the dimer stability, and at the same time, the salt bridges between A and B strands and the hydrophobic interactions are fundamental to ensure the dimer formation.

The dissociation can be described as a multi-step process. This is confirmed by an interdomain contact analysis during the domain separation, and from a kinetic and thermodynamic point of view. Figure 4 shows the change in contacts between loops/strands of the two domains during dissociation. The bound structure is characterized by interactions between the elements A, AB, B, CD, D, DE, E and G in both chains. In the process of dissociation, first the G-strands lose contact with the facing domains and the elements A, AB, B, CD, D, DE, E of both chains are still interacting. Then, the structure can dissociate completely, or it can still form another intermediate state, that is held together only by the β-strands D and E and the neighboring loop DE. The encounter complex then evolves into a fully dissociated state. All these shifts in contacts go hand in hand with a torsion of one domain against the other, reflected in the AB angle (Supplementary Table 2, Supplementary Fig. 6).

The three different states are also supported by a Markov state model, which we built for the wildtype (PDB code: 3AVE) (Fig. 5). The bound state of the wildtype is highly populated (97%) and evolves into the other two states in the microsecond timescale. The interdomain interaction energies confirm that the encounter complex and the undissociated state are less stabilized by VdW than electrostatic interactions, due to separation of the domains (Fig. 5c). Moreover, the analysis of the three states confirms that the biggest shift is present in the AB angle (Fig. 5d), and that the G-strand is the first one to lose contact with the facing chain in the encounter complex (Fig. 5e).

## Conclusions

We provided a detailed analysis of the dissociation of 19 C_H_3-C_H_3 crystal structures and a modelled one, to investigate the impact of interface mutations on this process. We found a correlation between the free energy of the bound states in our simulations and experimental melting temperatures of these systems. Furthermore, in agreement with previous literature results, we pointed out the relevance of several residues in the hydrophobic core of the interface for the stabilization of the domains. Moreover, we confirmed that the number of salt bridges is higher than the one of hydrophobic interactions in the bound state. We described the C_H_3-C_H_3 dissociation as a two-steps process, including a partly dissociated encounter complex. We highlight the β-strands that stabilize this intermediate state and the relevant residues that make contacts. Understanding the dissociation mechanism and the role of specific point mutations on the interface stability is crucial for the development of more innovative and developable therapeutics. Our computational approach can be generalized to other biomolecular interfaces to guide to the rational design of biotherapeutic proteins.

## Supplementary Material

Supplementary_File_gzac012

## Data Availability

The authors confirm that the data supporting the findings of this study are available within the article and its supplementary materials.

## Notes

Edited by: Professor Timothy Whitehead
