# Supplementary material for: Bispecific antibodies—effects of point mutations on CH3-CH3 interface stability
Source: Protein Eng Des Sel. 2022 Dec 5;35:gzac012. doi: 10.1093/protein/gzac012 (PMC9741699; doi:10.1093/protein/gzac012)
Supplement: Supplementary_File_gzac012 [file supplementary_file_gzac012.pdf]

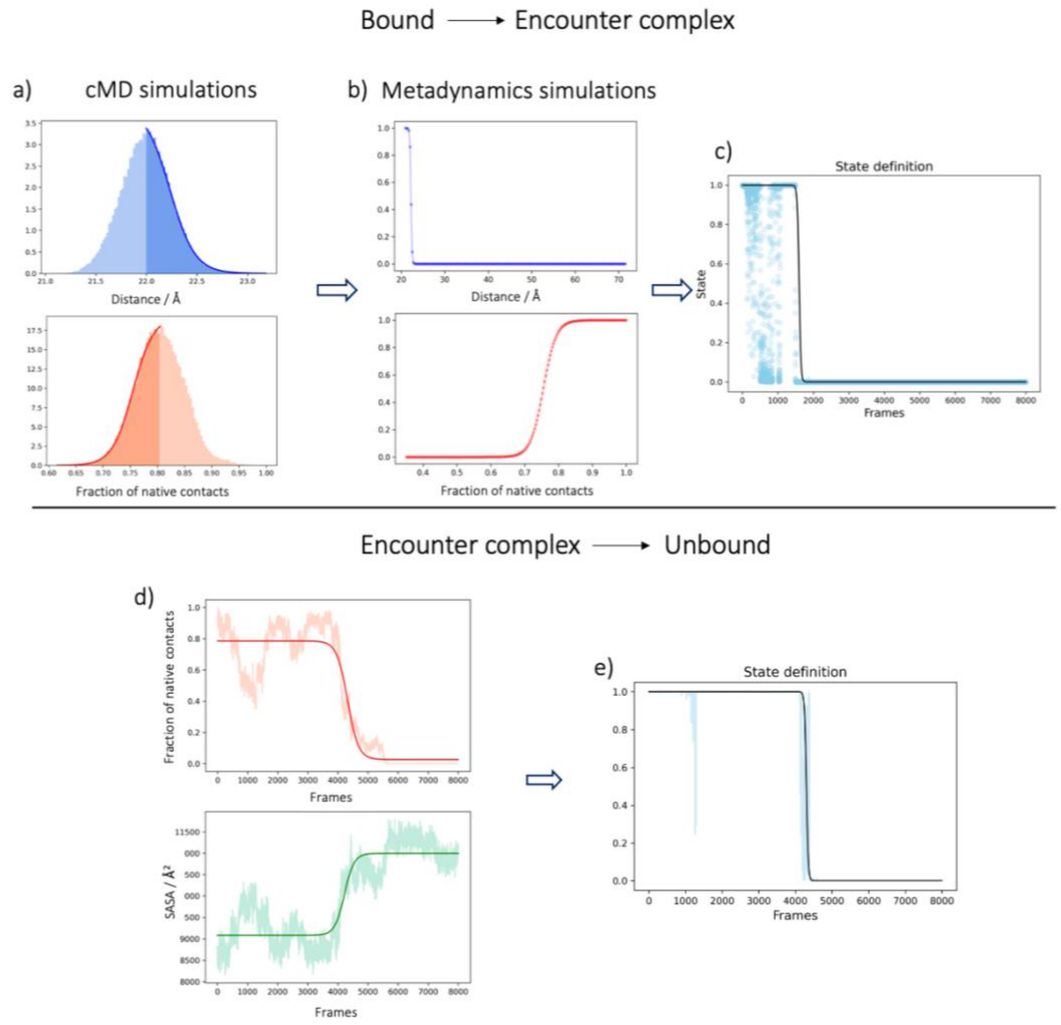

SI Fig.1. Definition of the dissociation state a) Representation of the Gaussian-shaped distribution of distance and fraction of native contacts during the cMD simulation, used as descriptors to define the dissociation points in the metadynamics simulations. A decreasing sigmoidal function is fitted on the right side of the distance distribution and an increasing one is fitted on the left side of the fraction of native contacts distribution. **b)** The parameters of the previous functions are used to plot new sigmoids on the values of distances and fraction of native contacts that are sampled during the metadynamics simulations. These sigmoids approach 1 when the structure is still bound, and they approach 0 when the dissociation starts. **c)** Time series of the state definition obtained by multiplication of the two sigmoids in **Fig.2b**. It shows the state of the system over time, starting from a bound structure (corresponding to 1), towards a dissociated state (0). A new sigmoidal function can be fitted to remove the noise. The point at which the sigmoid approaches 0 is considered the start of the dissociation process. **d)** To describe the transition to a completely unbound state, a sigmoidal function was fitted on the timeseries of the fraction of native contacts and SASA during the metadynamics

simulations. **e)** The normalization and multiplication of the two curves lead to the state definition plot. The curve can also be approximated by another sigmoid, to reduce the noise. When it approaches 1, the structure is still bound or in the process of dissociation. For values that are close to 0, instead, the structure can be considered completely dissociated.

SI Table 1. Residues that make contact in the first frame of the metadynamics simulations.

3AVE	
**chain a**	**chain b**
ASP399	LYS409
LYS409	ASP399
GLU357	LYS370
ASP356	LYS439
LYS370	GLU357
PHE405	LYS409
LYS409	PHE405
TYR407	TYR407
THR366	TYR407
LEU398	LYS392
GLU357	TYR349
TYR407	THR366

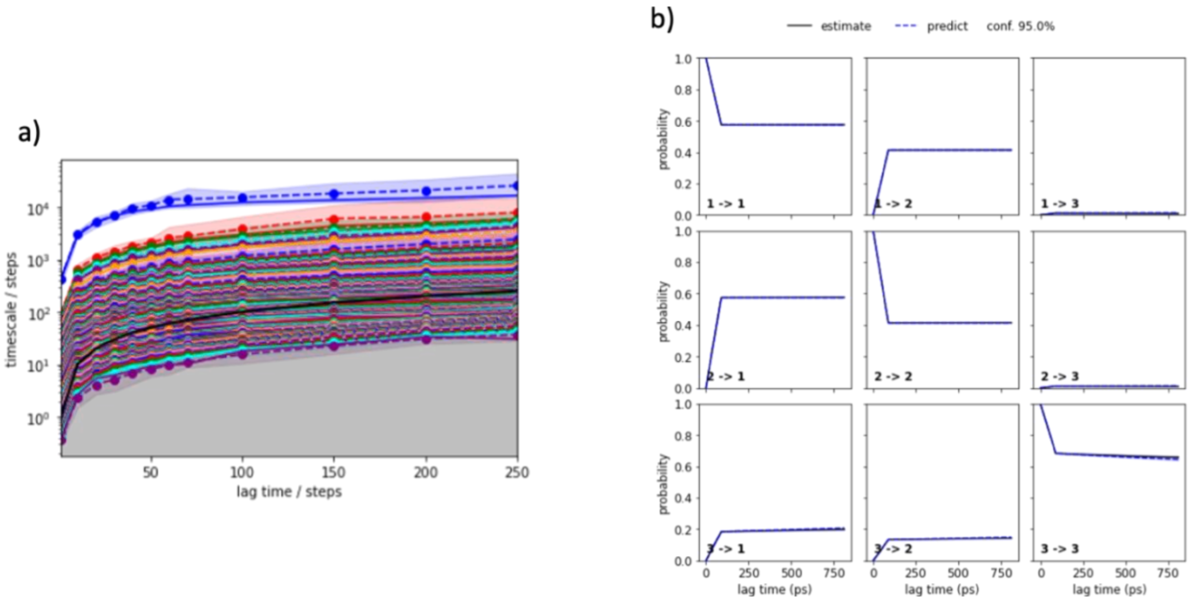

SI Fig.2. Validation of the Markov State Model. a) Implied timescale plot. Based on its convergence, we chose a lag time of 90 steps, correspondent to 9 ns. **b)** Chapman-Kolmogorov test for the evaluation of the Markov State Model, which shows good agreement between higher lagtime estimation and model prediction.

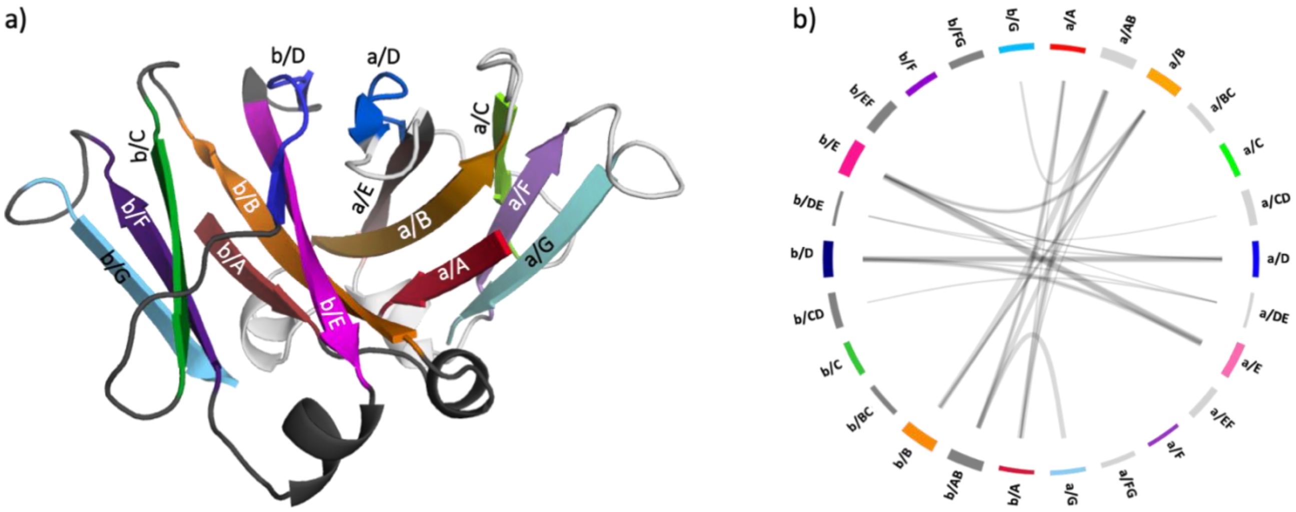

SI Fig.3. Coarse graining of the secondary structure elements. a) Schematic representation of a C_H3-C_H3 structure with coarse-grained loops and β-sheets. Chain A is on the right side and chain B on the left. The loops in chain A are colored in light grey, whereas the ones in the chain B are dark grey. **b)** Example of flareplot showing the contacts at the interface between chain A (on the left side) and chain B (on the right side). The residues belonging to the same secondary structure element are coarse grained together and color coded according to the structure in **SI Fig.3a**. The letters representing each loop or β-sheet are preceded by the letter "a_" if they belong to chain A or "b_" if they are in chain B.

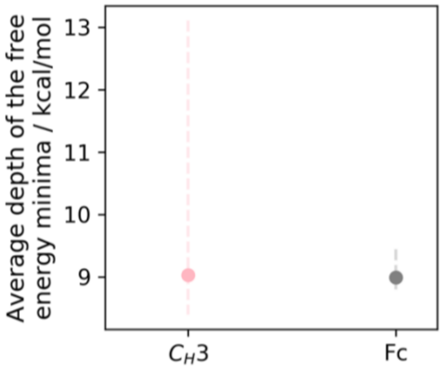

SI Fig.4 Comparison of the average free energy minima between the Fc fragment and the C_H3 dimer only. The points in the plot represent the average depth of the free energy minima for the C_H3 dimer and for the Fc fragment of a KIH system (PDB code: 4NQS). The error bars show the minimum and maximum of the average from the Leave-One-Out cross-validation.

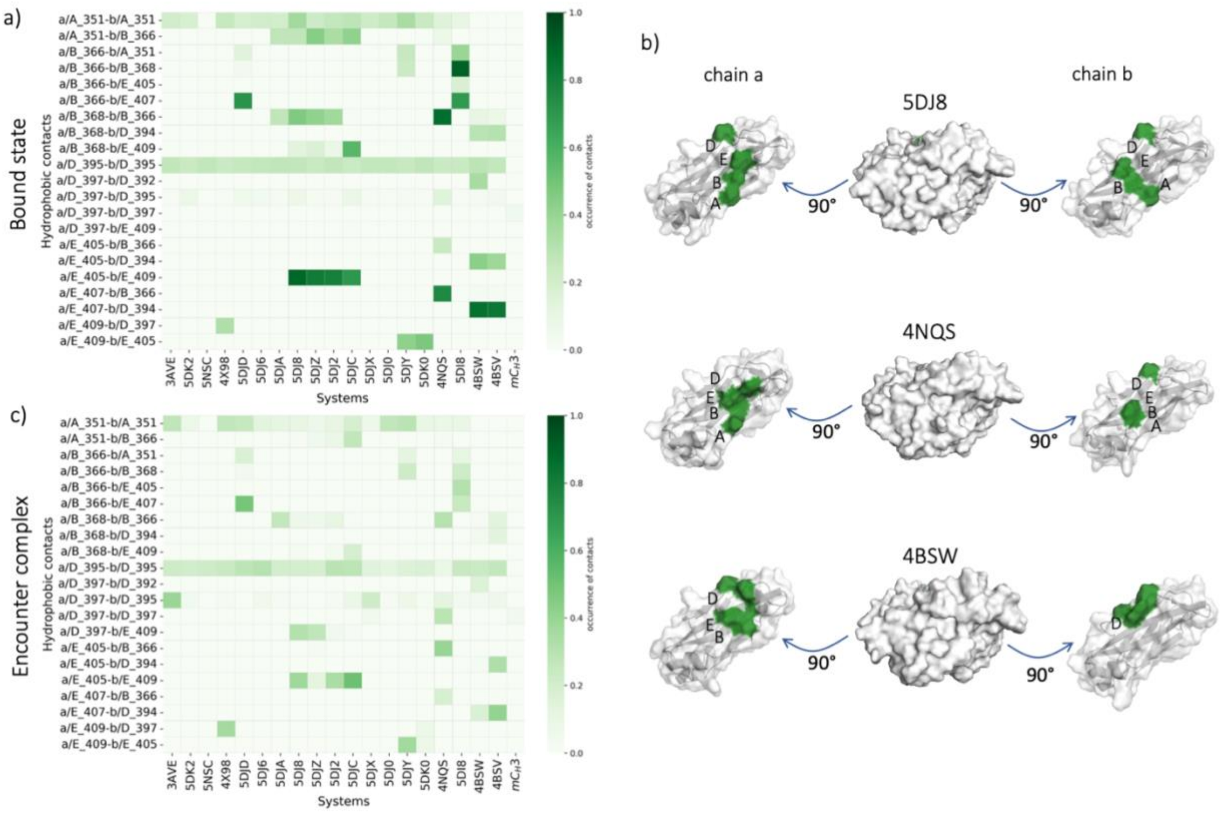

SI Fig.5. Comparison of hydrophobic contacts in the bound structures of the systems. a) Occurrence of hydrophobic contacts between chain a and chain b of all the system during the metadynamics simulations, until the start of the dissociation. The residues involved in a salt bridge are listed on the vertical axis of the matrix. The residue number is accompanied by a first letter (a or b), representing the chain, and a capital letter, representing the strand or loop in which the residue is located. **b)** The pictures show the open interface of the 5DJ8, 4NQS and 4BSW. The residues in the interface that make hydrophobic contacts are colored. **c)** Occurrence of hydrophobic contacts between chain a and chain b when the systems are in the encounter complex states, colored according to their occurrence.

	AB	AC1	BC1	AC2	BC2
3AVE	8.5	5.92	5.56	4.12	4.84
5DK2	10.52	7.88	7.02	6.6	8.79
5NSC	8.55	6.78	7.71	7.25	5.1
4X98	9.08	5.84	7.4	5.52	5.03
5DJD	5.94	5.47	6.68	4.12	4.71
5DJ6	10.0	7.37	6.4	5.03	4.54
5DJA	6.9	6.38	6.85	6.44	5.05
5DJ8	11.12	10.04	6.62	10.12	5.87
5DJZ	11.62	11.49	5.98	5.97	4.1
5DJ2	12.21	9.29	6.09	8.27	5.16
5DJC	7.69	6.6	5.24	7.21	4.7
5DJX	10.62	8.63	6.49	4.86	5.76
5DJ0	7.39	6.16	7.2	4.28	3.93
5DJY	10.05	6.32	8.1	5.32	6.11
5DK0	7.76	5.51	8.06	5.06	6.03
4NQS	7.91	6.82	7.1	5.9	5.64
5DI8	15.02	8.16	9.63	7.93	8.11
4BSW	11.2	8.38	9.68	6.27	8.54
4BSV	10.0	7.93	7.67	5.13	9.31

SI Table 2. Mechanism of dissociation via angles. The standard deviation of the angles during the 10 simulations for each system in the bound and encounter state have been averaged together and are presented in this table. For 17 systems, the highest deviation is present in the AB angle.

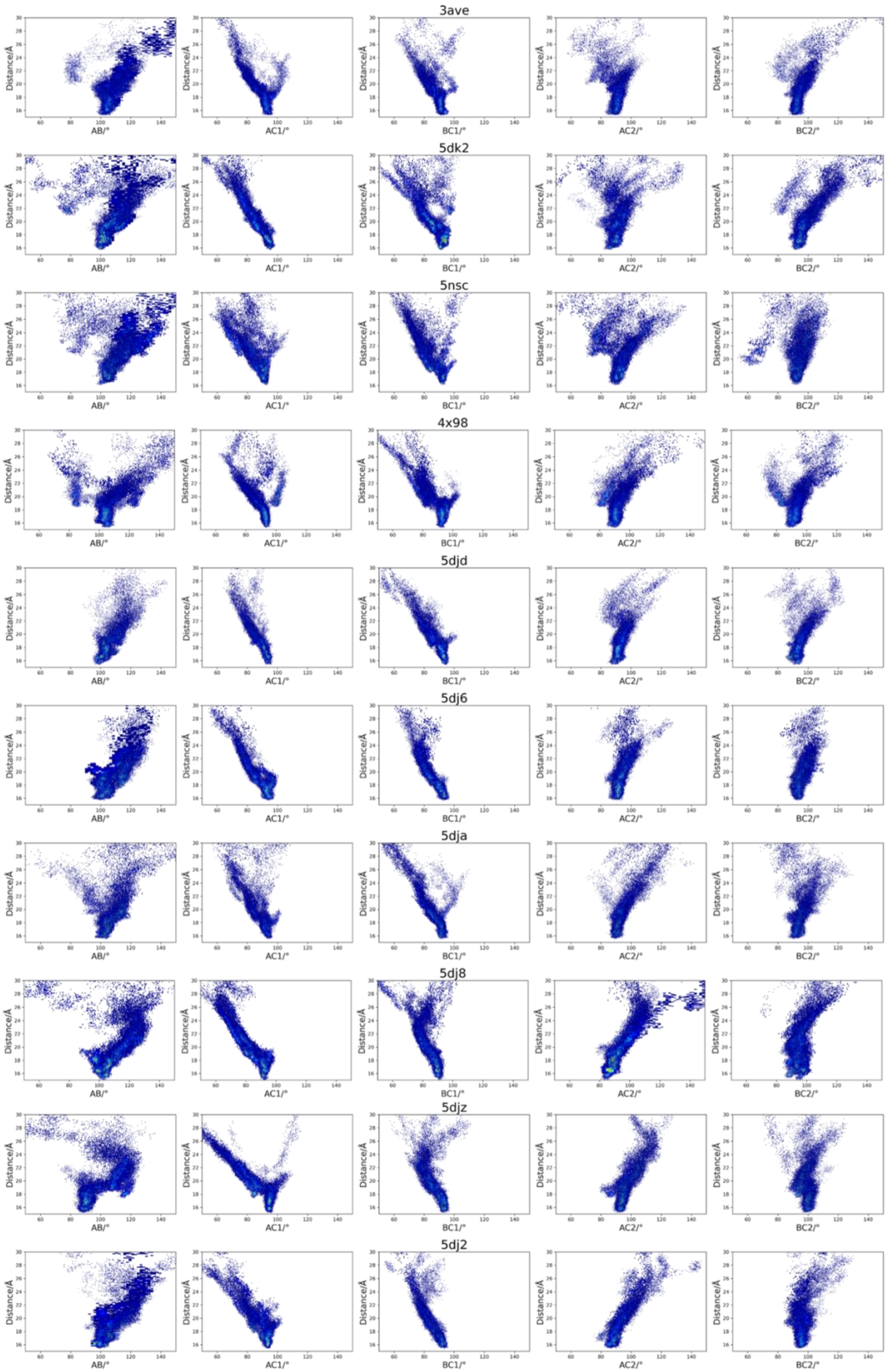
3ave
5dk2
5nsc
4x98
5djd
5dj6
5dja
5dj8
5djz
5dj2
Distance/Å
AB/°
AC1/°
BC1/°
AC2/°
BC2/°

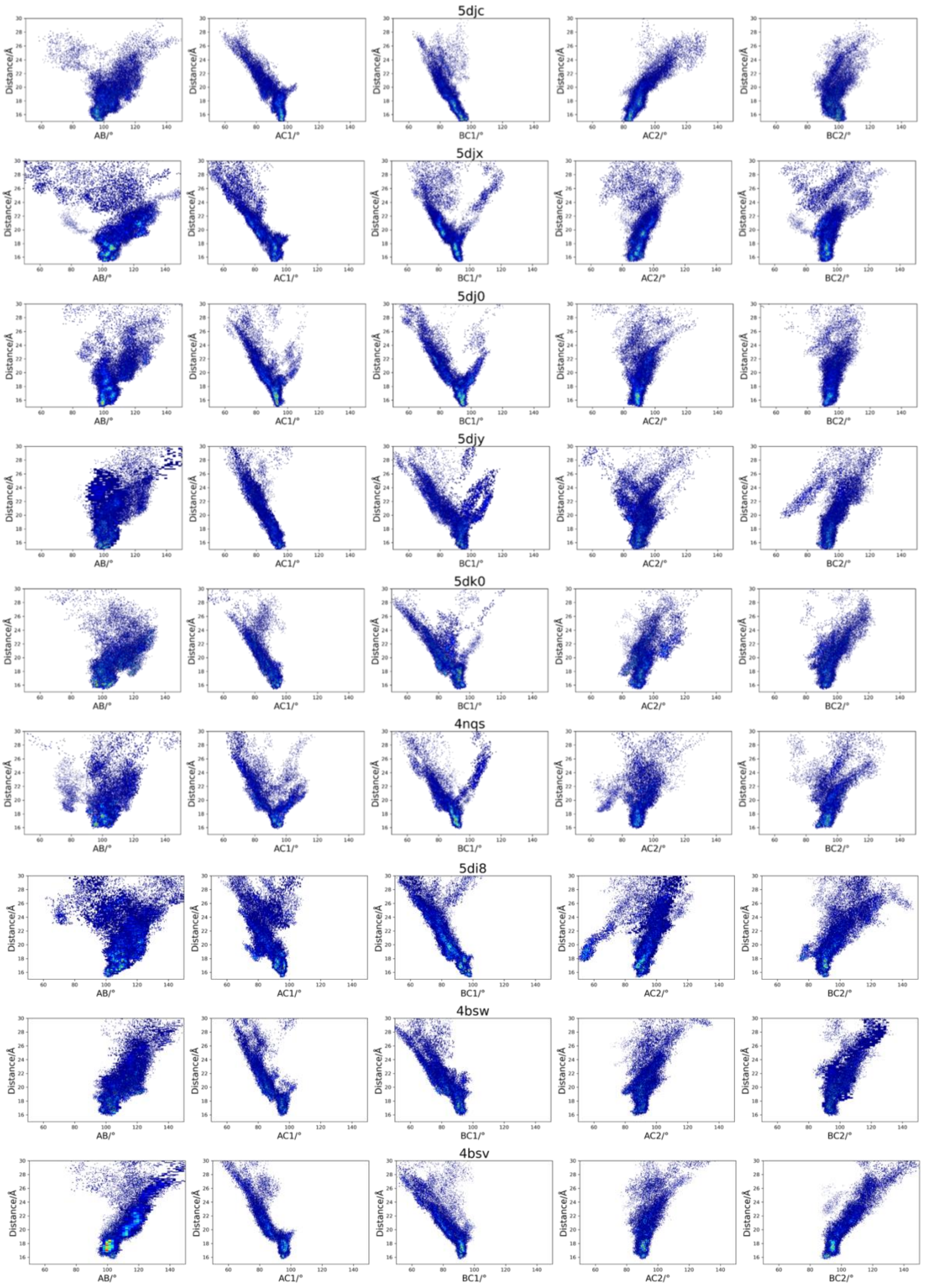

SI Fig.6 Shifts in the angles during dissociation. For each system, we represent the variation of each angle in relation to the distance, during the dissociation process. We considered only the

bound states and the encounter complexes during the metadynamics simulations and the ten repetitions of one system are merged.
